# How Molecular Typing Can Support *Legionella* Environmental Surveillance in Hot Water Distribution Systems: A Hospital Experience

**DOI:** 10.3390/ijerph17228662

**Published:** 2020-11-21

**Authors:** Luna Girolamini, Silvano Salaris, Jessica Lizzadro, Marta Mazzotta, Maria Rosaria Pascale, Tiziana Pellati, Sandra Cristino

**Affiliations:** 1 Department of Biological, Geological, and Environmental Sciences, University of Bologna, via San Giacomo 12, 40126 Bologna, Italy; luna.girolamini2@unibo.it (L.G.); silvano.salaris@unibo.it (S.S.); jessica.lizzadro2@unibo.it (J.L.); marta.mazzotta2@unibo.it (M.M.); mariarosaria.pascal2@unibo.it (M.R.P.); 2 GVM Care & Research, Lugo di Ravenna, 48022 Ravenna, Italy; tpellati@gvm-engineering.it

**Keywords:** *Legionellapneumophila*, non-*pneumophila Legionella* species, culture technique, sequence-based typing (SBT), macrophage infectivity potentiator (*mip*) sequencing, sequence types (STs), disinfection treatment, water safety plan

## Abstract

In this study, we aimed to associate the molecular typing of *Legionella* isolates with a culture technique during routine *Legionella* hospital environmental surveillance in hot water distribution systems (HWDSs) to develop a risk map able to be used to prevent nosocomial infections and formulate appropriate preventive measures. Hot water samples were cultured according to ISO 11731:2017. The isolates were serotyped using an agglutination test and genotyped by sequence-based typing (SBT) for *Legionella pneumophila* or macrophage infectivity potentiator (*mip*) gene sequencing for non-*pneumophila Legionella* species. The isolates’ relationship was phylogenetically analyzed. The *Legionella* distribution and level of contamination were studied in relation to temperature and disinfectant residues. The culture technique detected 62.21% of *Legionella* positive samples, characterized by *L. pneumophila* serogroup 1, *Legionella* non-*pneumophila*, or both simultaneously. The SBT assigned two sequence types (STs): ST1, the most prevalent in Italy, and ST104, which had never been isolated before. The *mip* gene sequencing detected *L. anisa* and *L. rubrilucens*. The phylogenetic analysis showed distinct clusters for each species. The distribution of *Legionella* isolates showed significant differences between buildings, with a negative correlation between the measured level of contamination, disinfectant, and temperature. The *Legionella* molecular approach introduced in HWDSs environmental surveillance permits (i) a risk map to be outlined that can help formulate appropriate disinfection strategies and (ii) rapid epidemiological investigations to quickly identify the source of *Legionella* infections.

## 1. Introduction

Legionnaires’ disease (LD) is caused by *Legionella* spp., which are environmental Gram-negative bacteria that colonize and persist in moist environments, particularly in water distribution systems [1]. Examples of water systems that might spread *Legionella* include hot and cold water tanks, heaters, large plumbing systems, cooling towers (air-conditioning systems for large buildings), medical devices (e.g., dental unit waterlines), and hot tubs, and unconventional sources such as decorative fountains, spray irrigation systems, and car washes [2,3]. The infection is acquired via inhalation of contaminated aerosol or, less commonly, by aspiration of drinking water [4]. 

The increasing incidence of both nosocomial and community-acquired *Legionella* infections has been a major public health concern. In 2018 in Italy, 2964 cases were reported to the National Surveillance System (2876 confirmed and 88 probable), with an incidence of 48.9 cases per million inhabitants, with a lethality rate for community and healthcare cases of 10.9% and 51.7%, respectively [5]. Among the 62 species of *Legionella* described to date, *Legionella pneumophila* serogroup 1 (SG1) is responsible for 75% of culture-confirmed LD cases [3].

The real risk of other sources of infection still remains somewhat underestimated. For this reason, appropriate *Legionella* risk assessment plans must be designed for water systems, as suggested by the Italian Guidelines [6], which will help minimize the risk of colonization, since eradication by water networks is impossible, particularly in the long term [7].

Many factors can enhance the risk of *Legionella* infection, such as the design, construction, and maintenance of water distribution systems, in addition to the presence of people who may be exposed, their vulnerability to infection, the degree of water system colonization (number of *Legionella* colonies, percentage of *Legionella* positive samples), and the pathogenicity of *Legionella* strains [8]. Each water distribution system should be assessed individually considering the building’s characteristics, the susceptibility of the population, and the modality of transmission from water sources. This assessment can be performed with the application of a water safety plan (WSP) approach that describes the most effective method to minimize the risk from poor water quality from the source to the point of use. This approach includes: (i) continuously updating the management plan to control risk; (ii) identifying a monitoring program; (iii) reviewing governance procedures including the management structure, responsibilities, and accountabilities of the individuals involved; (iv) establishing training requirements and measures of competence; and (v) constructing plans to deal with predictable events such as adverse results or cases associated with critical failures in the system (e.g., failure of major equipment such as a biocide dosing pump) [9].

Many studies have demonstrated the utility of performing *Legionella* typing to conduct epidemiological investigation, which is useful for establishing a link between environmental sources of infection and clinical cases, and for implementing appropriate risk control measures. Therefore, for epidemiological investigations, two properties of *Legionella* strains are usually determined: serological group (especially SG1) and genotype [8,10].

Molecular techniques can rapidly obtain information for identifying and genotyping the various species and serogroups of *Legionella*. Currently, there is no ideal genotyping method that is universally valid, since each organism appears to be better differentiated by one method over another. *Legionella* molecular typing is currently performed by sequence-based typing (SBT) for *L. pneumophila* strains and macrophage infectivity potentiator (*mip*) gene amplification for non-*pneumophila Legionella* species. These are useful tools during investigations of LD cases, clusters, or outbreaks [11,12]. 

The SBT technique is a variant of the multilocus sequence typing (MLST) schemes used to identify bacterial lineages and was first described for *Streptococcus pneumoniae* by Enright and Spratt [13], who used seven housekeeping genes. The SBT technique was developed by members of the European Legionnaires’ Disease Surveillance Network (ELDSNet) and has been described as a simple, rapid, discriminatory, and portable method for typing *L. pneumophila* strains [14]. The SBT technique is based on a scheme developed with a combination of seven housekeeping and virulence genes (*flaA, pilE, asd, mip, mompS, proA*, and *neuA*). The technique is now considered the gold standard for genotyping and is useful for identifying the sources of *L. pneumophila* infections, demonstrating the link between clinical and environmental isolates [15], although several studies have demonstrated its limitations. Generally, this approach is performed only during epidemiological investigations undertaken after the notice of a single case, cluster, or epidemic event. In contrast, environmental surveillance of *Legionella* is conducted using the culture technique, and in recent years, as suggested by Italian Guidelines [6], real-time polymerase chain reaction (RT-PCR) was introduced to confirm negative results or to rapidly screen water samples during epidemic events. 

Regarding non-*pneumophila Legionella* species, *mip* gene sequencing is useful in typing these species. The *mip* gene encodes a protein involved in the virulence of *Legionella* [16]. The *mip* gene sequence has been extensively studied, as it is extremely useful in typing studies; its presence in a single copy and its difference in species with a cut-off of 98% of homology helps to establish diversity between species, as suggested by the protocol and database of the European Working Group for *Legionella* Infections (EWGLI) [17], leading to unique identification.

The purpose of this study was to apply a genotyping approach during routine *Legionella* surveillance in a hospital hot water distribution system to assess *Legionella* contamination during a seven-year treatment with hydrogen peroxide (H_2_O_2_) and silver (Ag^+^) salts (H_2_O_2_/Ag^+^). The study focused on evaluating the changes in terms of positive samples, *Legionella* concentration, and isolate distribution. The genotyping approach and the analysis of phylogenetic relationships between strains enabled the study of the correlations or differences between strains and their response to physical–chemical parameters involved in *Legionella* ecology, such as temperature and disinfection treatment. The acquired knowledge permits a risk map of *Legionella* to be created for each building, which could support nosocomial infection control, maintenance and disinfection strategies of water distribution systems, as well as facilitate rapid response during epidemiological investigation by genomic comparison with clinical cases.

## 2. Materials and Methods 

### 2.1. Hospital Characteristics 

This study was conducted in an Italian hospital located in the Emilia-Romagna region that has implemented a WSP involving a *Legionella* surveillance program according to the Italian Guidelines [6]. The program consists of sampling the hot water distribution system every 3 months for a total of 4 samplings per year, following a risk assessment plan. The hospital structure consists of 3 separate buildings, buildings A, B, and C, with a total area of 32,194.60 m^2^ distributed as follows: 20,583.30, 10,111.3, and 1500 m^2^, respectively. Each building is supplied by the same municipal water, treated by a general softener to reduce the hardness to between 12–15°f (moderately hard), which is in line with Italian and European Council directives [18]. The water distribution system of the hospital complex consists of 3 heat exchangers that produce hot water with 3 hot water return line networks.

The risk assessment plan to control *Legionella* contamination applied in all hospital buildings consisted of sampling 5 sampling points in the technical rooms (aqueduct, tap water output, and 3 hot water return lines) and some of the 55 sampling points among common areas; consulting, diagnostic, and operating rooms; and offices, services, and inpatient rooms (located variously in the 3 buildings) every 3 months. All sampling points were identified in 3 locations: near, intermediate, and far from technical rooms, following the Italian Guidelines for *Legionella* prevention [6,7]. Despite the large number of inpatient rooms, the alternating sampling method enables sampling of almost all inpatient rooms in the 3 buildings. The layout of the sampling points, their locations on the floors, and their respective location in relation to the distance from technical rooms are shown in Table 1.

### 2.2. Hot Water Network Disinfection Treatment 

The control of *Legionella* contamination in the hospital complex started in 2013 with the installation of disinfection treatment based on a stabilized combination of H_2_O_2_ (34%, wt/wt) and Ag^+^ salts (0.003%, wt/wt) in demineralized water to increase the power of disinfection, according to Shuval et al. [15]. It is licensed by European and Italian legislations [19,20] for application to drinking water. The synergistic action of H_2_O_2_ and Ag^+^ salts makes the biocide more powerful than H_2_O_2_ alone [21,22]. The disinfectant is injected after hot water is output downstream from the heat exchangers (mixed water) and is dosed in proportion to water consumption. The concentration of disinfectant injected into the water supply of the 3 buildings is about 30 mg/L to obtain a concentration of about 5–10 mg/L at distal outlets.

### 2.3. Sample Collection

A total of 307 samples were collected from 2013 to 2019, and the number of samples were increased in relation to changes in hospital layout and the intended use of outlets. To assess the water quality in the main distribution system, 2 L samples of hot water were collected in post-flushing mode (running water for 1 min) in sterile polytetrafluoroethylene (PTFE) bottles containing a sodium thiosulphate solution (20 mg/L) [23]. The samples were stored in coolers (at about 4 °C), transported to the laboratory, and processed on the same day. During sampling, physical and chemical parameters (temperature and H_2_O_2_/Ag^+^) measured at outlets, maintenance procedures (e.g., disinfection procedure, thermostatic radiator valve, faucet replacement), and emergency servicing (e.g., shock disinfection treatment, increased temperature) performed on the hospital water system were recorded in a special register, as prescribed by Italian Guidelines.

### 2.4. Microbiological Analysis 

*Legionella* culture was performed in accordance with ISO 11731:2017 [24]. Each hot water sample was concentrated using polyethersulfone membrane filters with a porosity of 0.22 μm (Sartorius, Bedford, MA, USA). To quantify *Legionella* spp., aliquots of the samples (0.2 mL untreated and 0.1 mL filtered and heat- and acid-treated) were directly plated onto *Legionella* selective medium glycine–polymyxin B-vancomycin–cycloheximide (GVPC) plates (Thermo Fisher Scientific Diagnostic, Ltd., Basingstoke, UK). All plates were incubated aerobically at 35 ± 2 °C with 2.5% of CO_2_ for up to 15 days. Every 2 days, the plates were examined and presumptive colonies were counted and subcultured on buffered charcoal yeast extract (BCYE) agar with l-cysteine (cys+) and without L-cysteine (cys-) supplement (Thermo Fisher Scientific Diagnostic, Ltd., Basingstoke, UK). *Legionella* colony growth was observed on BCYE agar cys+, but not in BCYE cys-.

### 2.5. Serological Identification 

Five presumptive colonies for each plate were verified using a serological agglutination test (*Legionella* latex test kit, Thermo Fisher Scientific, Ltd., Basingstoke, UK) following the manufacturer’s instructions. This test allows the separate identification of *L. pneumophila* SG1 and SG2–14 and detection of seven other non-*pneumophila Legionella* species that are involved in human disease (*L. longbeachae* 1 and 2, *L. bozemanii* 1 and 2, *L. dumoffii, L. gormanii, L. jordani, L. micdadei,* and *L. anisa*). The isolates identified as *L. pneumophila* SG2 and 14 were then processed for single serogroup identification using polyclonal latex reagents (Biolife, Milan, Italy).

The data obtained are expressed as mean concentration ± SD and colony forming units (CFU)/L. According to the Italian Guidelines, the absence of *Legionella* in culture is expressed in relation to the volume of filtered water; therefore, the detection limit of the technique for 2 L samples is 50 CFU/L.

### 2.6. SBT Typing and Sequencing

Colonies identified by the agglutination test as belonging to the genus *Legionella* were subsequently analyzed by DNA sequencing. Genomic DNA was extracted from cultures using an InstaGene Purification Matrix (Bio-Rad, Hercules, CA, USA), and DNA concentrations were determined using a Qubit fluorometer (Thermo Fisher Scientific, Paisley, UK). We selected 26 strains identified as *L. pneumophila* as representative of all samples and these were analyzed by SBT to determine the sequence type (ST). SBT using *flaA*, *pilE*, *asd*, *mip*, *mompS*, pro, and *neuA* loci was performed according to the ELDSNet protocol (Appendix 1) [25].

Genotype analysis was based on the sequencing of all 7 genes. PCR products were visualized by electrophoresis on 2% agarose gel and staining with ethidium bromide. Amplicons obtained for each of the seven genes after purification were subjected to a sequencing reaction cycle. Each purified PCR fragment was subjected to two cyclic linear polymerization reactions (one for sequencing the filament forward (Fw) and one for the filament reverse (Rw)) using tailed primers Fw-M13 and Rw-M13. Following purification, the product of cycle sequencing was subjected to capillary electrophoresis in an automated system for fluorescence (ABI PRISM 3100 Genetic Analyzer, Applied Biosystems, Foster City, CA, USA) with a laser beam that is capable of exciting the four fluorophores. The nucleotide sequences obtained were confirmed by the SBT database, available on the EWGLI website (http://www.ewgli.org/), and the sequences were compared with those in the ELDSNet database to assign the ST allelic profile (http://www.hpa-bioinformatics.org.uk/legionella/legionella_sbt/php/sbt_query_frontpage.php).

### 2.7. mip Gene Sequencing

The strains serotyped by agglutination as non-*pneumophila Legionella* species were identified by analyzing the *mip* gene sequence using bacterial DNA purified from isolated colonies. *mip* was amplified with PCR using degenerate primers, as described by Ratcliff et al. [26], and modified using M13 tailings to avoid noise in the DNA sequence [26,27]. Gene amplification was performed in a 50 µL reaction containing DreamTaq Green PCR Master Mix 2 (Thermo Fisher Scientific Inc., Waltham, MA, USA) and 40 pM of each primer; 100 ng of DNA extracted from the presumptive colonies of non-*pneumophila Legionella* species was added as template. The protocol used for this purpose was developed and standardized by EWGLI (changed in 2011 by ESGLI), the sequences obtained are comparable to those available in the database (http://www.hpa.org.uk/cfi/bioinformatics/dbases.htm# EWGLI), and return the identity for isolates belonging to *Legionella* species (Appendix 2, Ratcliff protocol). PCR products were visualized by electrophoresis on 2% agarose gel, staining with ethidium bromide. Following purification, they were sequenced using BigDye Chemistry and analyzed on an ABI PRISM 3100 Genetic Analyzer (Applied Biosystems, Foster City, CA, USA). Specifically, *mip* amplicons were sequenced using tailed M13 forward and reverse primers (mip-74F-M13F tgtaaaacgacggccagtGCTGCAACCGATGCCAC; mip-595R-M13R caggaaacagctatgaccCATATGCAAGACCTGAGGGAAC) to obtain complete coverage of the sequenced region of interest [27].

Raw sequencing data were assembled using CLC Main Workbench 7.6.4 software (Qiagen, Redwood City, CA, USA). The sequences were compared with the reference ones deposited in the *Legionella mip* gene sequence database using a similarity analysis tool (http://bioinformatics.phe.org.uk/cgi-bin/Legionella/mip/mip_id.cgi).

### 2.8. Phylogenetic and Allelic Diversity Analysis

Starting from the pherograms obtained by Sanger sequencing, phylogenetic analysis was performed by a Geneious Prime genome browser (Geneious Prime 2020.1.2; http://www.geneious.com) [28] for both *L. pneumophila* species (SBT sequences) and non-*pneumophila Legionella* species (*mip* gene). Multiple sequence alignments were carried out with the Geneious algorithm, which is a progressive pairwise aligner. From the nucleotide alignments, phylogenetic trees were inferred with FastTree based on heuristic neighbor joining and the Jukes–Cantor distance model. To quickly estimate the reliability of each split in the tree, FastTree uses the Shimodaira–Hasegawa test on three alternate topologies (nearest-neighbor interchanges (NNIs)) around that split. FastTree uses 1000 resamples and does not reoptimize the branch lengths for resampled alignments [29]. The sequences were trimmed to the correct length using the reference sequence specific to each allele, provided by EWGLI.

Regarding the SBT analysis, the seven genes were subsequently assembled to obtain a concatenated sequence with a length of 2501 bp. The concatenated SBT sequences were used for phylogenetic analysis. 

### 2.9. Statistical Analysis

Statistical analysis was performed using R Statistical Software (version 3.6.3, “Holding the Windsock” R Foundation for Statistical Computing, Vienna, Austria). The normality of variables was assessed using the Shapiro–Wilk test. The data were evaluated using Kruskal–Wallis and Mann–Whitney tests. Spearman’s rho rank correlation was calculated for all possible pairwise combinations. The significance of all statistical tests was set at *p* ≤ 0.05. 

## 3. Results

### 3.1. Microbiological, Physical, and Chemical Results

Hot water samples (*n* = 307) were analyzed for detection and enumeration of *Legionella* spp. 

The hospital complex showed different distributions of *Legionella* regarding the percentage of positive samples and mean *Legionella* level. The water reservoir and water output outlets were always *Legionella*-free (under the limit of the culture technique of <50 CFU/L of water).

The samples were distributed in the buildings as follows: 127 in building A, 122 in building B, and 58 in building C. The culture method identified 191/307 (62.2%) positive samples with a mean concentration ± standard deviation (SD) of 3562.43 ± 20,648.43 CFU/L. The percentage of positive samples with mean *Legionella* concentration was distributed in the three buildings as follows: 102/127 (80.31%) with a mean concentration of 7434.23 ± 31,459.66 CFU/L in building A, 48/122 (39.34%) with a mean concentration of 1066.80 ± 4520.13 CFU/L in building B, and 41/58 (70.69%) with a mean concentration of 333.93 ± 613.4 CFU/L in building C.

During the study, temperature and disinfectant residues were measured for all outlets sampled. The mean temperature was 49.36 ± 2.61 °C, while the mean amount of the disinfectant was 12.32 ± 5.35 mg/L. In addition, the chemical water characteristics of the main cold and hot water distribution systems outlets such as the water reservoir, water output outlets, and hot water return lines of each building were measured during the study. The results are provided in Table S1. 

### 3.2. Legionella Serotyping and Genotyping Results

According to ISO 11731:2017 [24], at least five representative colonies of each type of subculture were confirmed by the agglutination test and were identified as *L. pneumophila* SG1 in 67/191 samples (35%) and non-*pneumophila Legionella* species in 41/191 samples (21.4%). In other samples, they were found together (83/191, 43.4%). 

The SBT analysis performed on isolates already identified as *L. pneumophila* SG1 assigned two STs, ST1 and ST104, with the following profiles: ST1 (1, 4, 3, 1, 1, 1, 1) and ST104 (3, 10, 1, 1, 14, 9, 1). ST1 is the most frequent isolate in Italy [30]. The ST104 isolate has not been previously reported in Italy; therefore, its profile was submitted to the EWGLI database with accession number EULV13742.

The *mip* gene sequencing identified, within non-*pneumophila Legionella* species, *L. anisa* and *L. rubrilucens*.

**Figure 1:**
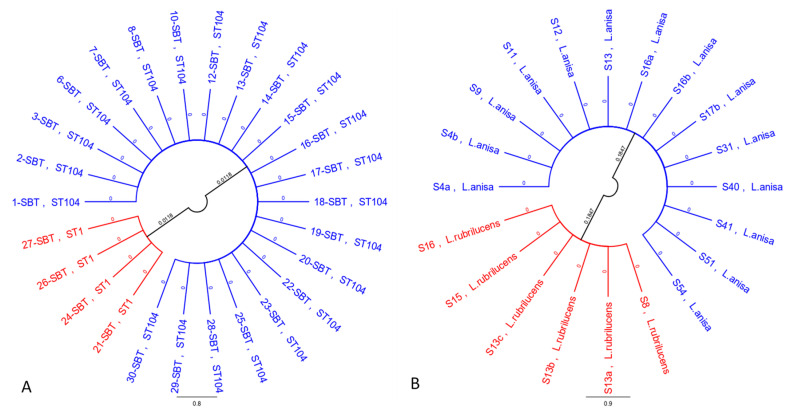
Phylogenetic trees of *L. pneumophila* (**A**) and non-*pneumophila Legionella* species (**B**) strains.

Multiple sequence alignment (MSA) and phylogenetic tree analysis for the strains subjected to SBT (*L. pneumophila*) distinguished the isolates in two groups, characterized by 97.7% nucleotide identity between them and 100% intracluster homology, with the same allelic profile for all seven genes (Figure 1A). The scale bars and values shown in Figure 1 indicate branch length in number of substitutions per site.

Phylogenetic analysis of the *mip* gene for non-*pneumophila Legionella* species determined the creation of a tree that clearly shows the genetic distance between *L. anisa* and *L. rubrilucens*, forming a distinct cluster for each species (Figure 1B). The two clusters shared 70.1% identity. 

### 3.3. Legionella Contamination in the Hospital 

The contamination analysis resulted in finding 191 positive samples within the total 307 samples. These data showed significant differences in terms of mean *Legionella* contamination between buildings, with building A being more contaminated than B and C. The differences found between buildings are presented in Table 2.

Building A had the highest levels of contamination, which were significantly different compared to buildings B and C. Building B was the least contaminated in terms of both *L. pneumophila* and non-*pneumophila Legionella* species.

The previous results were also analyzed considering mean concentration of *Legionella* isolates in the entire hospital complex and successively within each building. The results of comparison and the statistical analysis are summarized in Table 3.

Considering *Legionella* contamination in the hospital complex, we found a significant difference between *L. pneumophila* and non-*pneumophila Legionella* species (*p* = 5.67 × 10^−3^). The same trend was found in buildings B and C (*p* = 0.014 and *p* = 2.90 × 10^−3^, respectively).

A different *Legionella* strain distribution was found in each building regarding the percentage of positive samples and mean concentration throughout the observation period. From 191 positive samples, some samples were contaminated only by *L. pneumophila* or non-*pneumophila Legionella* species. In some cases, both species were present in the same sample; in this case, the contamination was labelled “both species”. The percentage of positive samples with mean *Legionella* contamination, temperature, and disinfectant residues measured are reported in Table 4. 

The data show that building A was more contaminated than the other buildings, especially regarding samples where both species were found. A comparison among all species typed in the hospital complex and in each building is shown in Table 5.

The results obtained regarding the typing and distribution of *Legionella* isolates were used to elaborate a risk map for each floor of the hospital complex. For each floor, the buildings are represented as follows: building A, blue square; building B, yellow square; and building C, green square. The map is shown in the Supplementary File (Figures S1 and S2).

### 3.4. Correlation between Legionella and Physical–Chemical Parameters

To correlate the microbiological results with other measured parameters, such as temperature and disinfectant residues, Spearman’s rho correlation tests were used for all possible pairwise combinations (*L. pneumophila,* non-*pneumophila Legionella* species, temperature, and disinfectant). 

In the 191 positive samples, a significant direct correlation was found between *L. pneumophila* and non-*pneumophila Legionella* species (*p* = 1 × 10^−5^). In contrast, a significant inverse correlation was found for both species and temperature; an increase in temperature led to a decrease in mean *Legionella* concentration (*p* = 1 × 10^−5^). Both species indirectly responded to temperature, and increased temperature has a negative impact on bacteria, according to previous findings [31]. Regarding the effect of disinfectant on *Legionella*, we found a significant inverse correlation (*p* = 4 × 10^−4^) with *L. pneumophila* where increased disinfectant dosage caused a decrease of *L. pneumophila* concentration. The same trend was found for non-*pneumophila Legionella* species, although non-significant data were obtained (*p* = 0.22). 

We also studied the correlation between *Legionella* contamination, temperature, and disinfectant for the hospital complex and within each building. The trend observed mimicked the general trend described above, although with a lack of significant data in a few cases. Table 6 summarizes all correlations between *Legionella*, temperature, and disinfectant. The correlation matrices obtained are shown in Supplementary Figures S3–S6.

## 4. Discussion

In this study, *Legionella* was environmentally surveilled by combining the standard culture method with molecular techniques, and innovative results were obtained, especially with regards to changes in the dynamics of colonization and the construction of a phylogenetic map that can support epidemiological investigations.

The culture technique is still considered the gold standard for quantification of *Legionella*, although it has several drawbacks, including long incubation time and poor sensitivity, causing delays in response times, especially during outbreaks of LD. Furthermore, it is unable to detect viable but nonculturable cells (VBNCs) [32], and the discrepant results between the culture method and PCR are most pronounced for non-*pneumophila Legionella* species [33].

The ability of public health organizations to respond rapidly to LD outbreaks is thus hampered due to the time required for culture. The aim of the microbiological technique during epidemiological investigation is to correlate the source of the outbreak with the cases, comparing *Legionella* isolates from patients with those from environmental samples. The identification of cases with a common source of infection is possible with accurate discrimination of *Legionella* isolates [34]. 

In this study, a molecular approach was combined with a culture technique in an Italian hospital during routine environmental monitoring. The hospital that was the subject of this study is organized in three separate buildings that use the same municipal water supply and three hot water return lines, which allowed a simultaneous and comparative study among the buildings. The introduction of a new disinfectant based on H_2_O_2_/Ag^+^ in 2013 enabled the control of the level of contamination in relation to disinfectant dosage and creating a new water safety plan compliant with directives, as demonstrated by our previous data [35].

Monitoring the hospital for seven years provided detailed insight into *Legionella* communities in addition to differences in the numbers of positive samples and contamination levels within each building. The results observed in each building included the percentage of positive samples and the level of *Legionella* contamination. Considering the percentage of positive samples, building A was more contaminated, followed by C and B. Regarding the mean level of contamination, building A remained the most contaminated, followed by B and C. Significant differences in *Legionella* types were found, with *L. pneumophila* being the predominant species. According to the Italian Guidelines, considering these two aspects (mean and percentage of positive samples) is important for controlling contamination, following the reference risk levels shown in the Guideline’s Table 11 [6]. These data can be explained by building A having a larger surface area and lower water demand compared with the other buildings, as indicated by water consumption (938.98 m^3^/year for building A, 1745.44 m^3^/year for building B, and 450.62 m^3^/year for building C). The low water demand of building A is due to work areas such as offices and consulting rooms being closed during weekends and holidays, so daily water flushing is low, which can influence water stagnation and the biofilm community [35,36].

The genotyping approach is usually applied only during epidemiological investigations. The SBT technique allowed us to infer the population structure of *L. pneumophila*, to study genetic diversity and clonal expansion, and to undertake long-term epidemiological analyses of microbial populations [37]. This approach was developed to compare clinical and environmental isolates, but in our study, we applied it to the environmental isolates that we found. 

The results showed that all strains belonging to *L. pneumophila* SG1 were typed as ST1 and ST104, with 100% homology in seven allelic gene profiles. ST104 has never been documented in Italy; therefore, our study proves its presence in Italy for the first time, and demonstrates how genotyping can identify the presence of new STs in the hospital environment, despite its occurrence during environmental surveillance.

Regarding *Legionella* non-*pneumophila* species, the isolates were typed as *L. anisa* and *L. rubrilucens. L. anisa* is well documented in the literature as a causative agent of different cases or epidemic events [38,39,40]. *L. rubrilucens* has been less studied, with few clinical cases reported and its pathogenicity has not been fully demonstrated [41].

The sequences of non-*pneumophila Legionella* species underwent phylogenetic analysis, showing the presence of two clusters, one formed by *L. rubrilucens* and one by *L. anisa*, confirming the genetic diversity of the two species. Genotyping data are useful for understanding the relationship between *Legionella* species in a hospital complex and in buildings, contributing to rapid epidemiological investigations, where matching clinical and environmental isolates is the critical step in identifying the reservoir of infection. The knowledge of strain characteristics could support preventive technical and maintenance procedures to control the risk of *Legionella* proliferation, including the choice of disinfectant, managing a developed strain’s resistance, controlling bacteria proliferation through temperature, and planned maintenance procedures (e.g., flushing of water pipelines to reduce dead branches). 

We found that *L. pneumophila* was significantly predominant in the hospital hot water distribution system compared to non-*pneumophila Legionella* species. This trend was observed in the entire hospital complex and in buildings B and C, but not in building A. The direct correlation (*p* = 1 × 10^−5^) between *L. pneumophila* and non-*pneumophila Legionella* species in the same samples indicates their cohabitation in the hospital environment, in line with the findings of Van Der Mee-Marquet [42]. To better understand the relationships between *Legionella* species, we correlated the mean contamination found with the physical and chemical parameters measured (temperature and disinfectant residues). Our data confirmed that *Legionella* is negatively controlled by temperature; increased temperature decreases *Legionella* concentration according to previous studies [36,43]. The mean temperature of the hot water in the hospital complex is close to 50 °C, and although some oscillations of 2–3 °C occur in the buildings, the water temperature seems to be the discriminant factor regarding the level of contamination, having a significant impact on *Legionella* control within buildings, irrespective of *Legionella* species. The correlation of disinfectant with *Legionella* species was significant and negative only for *L. pneumophila* in the hospital complex. This correlation was maintained in building B; in contrast, it lost significance in buildings A and C, although the trend was maintained. It seems that disinfectant works well to control *L. pneumophila*, but no significant correlation was found with non-*pneumophila Legionella* species, despite the negative correlation. 

Consulting the hospital maintenance register, we found two shock treatments performed in buildings A and C during the study, where H_2_O_2_/Ag^+^ increased up to 50 mg/L. This treatment may have influenced *L. pneumophila* more than non-*pneumophila Legionella* species, leading to an increase in non-*pneumophila Legionella* species alone or in samples with both species. 

These data are supported by those reported by Perrin et al., who demonstrated that some physical–chemical parameters have a different impact on bacteria [44]. In water distribution systems, in addition to ecological parameters (e.g., nutrients, the presence of protozoa or bacteriophages), disinfectants play an important role as abiotic regulators [45]. Non-*pneumophila Legionella* species could develop resistance to the disinfection treatment in line with the antibiotic effect and antibiotic resistance developed by other bacteria. Changes to the disinfectant dosage must consider these observations, especially in hospitals where immunocompromised patients may be exposed to other species that can be selected by the treatment, but are less associated with human disease. The focus of our future research will involve studying the response of *Legionella* species to different disinfectant concentrations and temperatures to find differences in terms of the sensitivity or resistance developed in a hot water distribution system.

We want to underline that serological identification, as a routine approach, provides a definition of *Legionella* with a limit of differentiation only between *L. pneumophila* (some serogroups) and non-*pneumophila Legionella* species (present in the agglutination latex test). This limit does not support the study of the dynamics of colonization and microbial diversity, especially in the presence of disinfection treatment, with underestimation occurring during culture. 

The approach used in this study is innovative for routine environmental monitoring as the directives require implementation of corrective measures based on the percentage of positive outlets and the level of contamination, regardless of the type of *Legionella* species found. 

Based on epidemiological data, the common opinion is that *L. pneumophila* SG1 is the main organism responsible for clinical cases, not non-*pneumophila Legionella* species. For this reason, preventive strategies or extraordinary measures are usually only undertaken when microbiological data indicates contamination by *L. pneumophila* [46]. Our approach promotes the study of the role of other *Legionella* species, which are often considered less important in terms of infection, although they may play a minor or unknown pathogenic role. We think that other species of *Legionella* that usually are less often detected than *L. pneumophila* and without clinical evidence could, in some conditions (e.g., absence of competition, changes in disinfectant dosage or water characteristics), develop a new pathogenic pattern, especially given our poor knowledge of all genes and protein effectors involved in *Legionella* pathways.

Our results show that the molecular approach could provide relevant data on species isolated by techniques other than culture: (i) by identifying the presence of a new ST, ST104, for the first time in Italy and (ii) by discriminating species within non-*pneumophila Legionella* species, also helping to understand (iii) the interaction between *L. pneumophila* and non-*pneumophila Legionella* species and (iv) the interaction between *Legionella* species and temperature and disinfectant. Our method allows (v) the possibility of constructing a map of *Legionella* contamination for each outlet monitored, providing complete knowledge of the *Legionella* distribution. 

## 5. Conclusions

Knowledge of *Legionella* strains can help health authorities, engineers, and technical staff to implement the correct measures to formulate preventative strategies. Our study confirms the role of the SBT technique and *mip* sequencing in studying the distribution of *Legionella* strains in environmental as well as in clinical surveillance to correctly establish the epidemic sources of infection, plan a long-term prevention strategy, and establish the correlations between isolates. 

One of the future goals could be to improve studies and techniques for typing non-*pneumophila Legionella* species that are less known and less associated with human disease through a whole genome sequence (WGS) approach. This would improve the knowledge of the pathogenicity, resistance, and ecological status of *Legionella* isolates that are often difficult to isolate and recognize during routine culture techniques, leading to an underestimation of the real risk of *Legionella* infection. 

In conclusion, in our opinion, good knowledge of species and strains that colonize water distribution systems is epidemiologically relevant as it supports the rapid implementation of interventions by hospitals or other facilities in clinical cases and avoids the waste of time and economic resources.

## Figures and Tables

**Table 1:** Distribution of sampling points within buildings and in technical rooms.

**Hospital Outlet Distribution**											
				**Sample ID**	**Sample Point**	**Location**					
				56	Aqueduct	Technical room					
				64	Tap water output	Technical room					
**Building A outlet distribution**				**Building B outlet distribution**				**Building C outlet distribution**			
**Sample ID**	**Sample Point**	**Floor**	**Location**	**Sample ID**	**Sample Point**	**Floor**	**Location**	**Sample ID**	**Sample Point**	**Floor**	**Location**
1a	Hot water return line building A	-	Technical room	1b	Hot water return line building B	-	Technical room	1c	Hot water return line building C	-	Technical room
1	Service	0	near	22	Service	−1		43	Service	−1	near
2	Common area	0	intermediate	23	Common area	0	near	44	Service	−1	far
3	Common area	0	far	24	Service	0	intermediate	45	Common area	0	near
4	Common area	0	far	25	Common area	0	intermediate	46	Service	0	far
5	Common area	1	near	26	Service	0	far	47	Service	1	near
6	Common area	1	intermediate	27	Operating room	0	far	48	Service	1	far
7	Service	1	far	28	Inpatient rooms	1	near	49	Common area	2	near
8	Common area	1	far	29	Service	1	intermediate	50	Inpatient rooms	3	near
9	Inpatient rooms	2	near	30	Inpatient rooms	1	intermediate	51	Inpatient rooms	3	intermediate
10	Service	2	intermediate	31	Inpatient rooms	1	far	52	Inpatient rooms	3	far
11	Inpatient rooms	2	far	32	Common area	1	far	53	Inpatient rooms	4	near
12	Operating room	2	far	33	Inpatient rooms	2	near	54	Inpatient rooms	4	intermediate
13	Operating room	2	far	34	Inpatient rooms	2	intermediate	55	Inpatient rooms	4	far
14	Intensive care	4	near	35	Service	2	intermediate				
15	Intensive care	4	intermediate	36	Inpatient rooms	2	far				
16	Intensive care	4	far	37	Inpatient rooms	2	far				
17	Common area	4	far	38	Inpatient rooms	3	near				
18	Operating room	5	near	39	Inpatient rooms	3	intermediate				
19	Common area	5	intermediate	40	Inpatient rooms	3	far				
20	Operating room	5	far	41	Inpatient rooms	4	near				
21	Operating room	5	far	42	Service	4	far				

**Table 2:** Comparison of mean Legionella concentration between buildings.

*Legionella* Species Isolated	Building Comparisons	Kruskal–Wallis Test *p*-Values	Type of Comparison	Mann–Whitney Test *p*-Values
Total *Legionella*	A ≠ B	2 × 10^−11^	A > B	3.26 × 10^−12^ *
	A ≠ C	6.32 × 10^−3^	A > C	3.16 × 10^−3^ *
	B ≠ C	2.20 × 10^−4^	B < C	7.45 × 10^−5^ *
*L. pneumophila* (SG1)	A ≠ B	2.60 × 10^−5^	A > B	4.36 × 10^−6^ *
	A ≠ C	0.535		
	B ≠ C	2.00 × 10^−3^	B < C	6.71 × 10^−4^ *
Non-*pneumophila Legionella* species	A ≠ B	5.10 × 10^−10^	A > B	8.56 × 10^−11^ *
	A ≠ C	1.60 × 10^−3^	A > C	5.27 × 10^−4^ *
	B ≠ C	8.50 × 10^−3^	B < C	4.27 × 10^−3^ *

* *p* ≤ 0.05.

**Table 3:** Comparison of means between *L. pneumophila* and non-*pneumophila Legionella* species in buildings.

Building	*Legionella* Comparison	Mann–Whitney Test *p*-Value	*Legionella* Comparison	Mann–Whitney Test *p*-Value
Hospital complex	*L. pneumophila* ≠ non-*pneumophila Legionella* species	0.011 *	*L. pneumophila* > non-*pneumophila Legionella* species	5.67 × 10^−3^ *
A	*L. pneumophila* ≠ non-*pneumophila Legionella* species	0.64		
B	*L. pneumophila* ≠ non-*pneumophila Legionella* species	0.027 *	*L. pneumophila* > non-*pneumophila Legionella* species	0.014 *
C	*L. pneumophila* ≠ non-*pneumophila Legionella* species	5.70 × 10^−3^ *	*L. pneumophila* > non-*pneumophila Legionella* species	2.90 × 10^−3^ *

* *p* ≤ 0.05.

**Table 4:** Isolate distribution between buildings and physical–chemical parameters measured.

Building	Sample Contamination	Number of Positive Samples, *n* (%)	*Legionella* Contamination, Mean ± SD (Log CFU/L)	Temperature, Mean ± SD (Minimum–Maximum) (°C)	H_2_O_2_ Residues, Mean ± SD (Min–Max) (mg/L)
Hospital complex	Total *Legionella*	191/307 (62.21%)	3562.43 ± 20,648.43	49.36 ± 2.61 (32.5–65.0)	12.24 ± 5.18 (0–25)
	Only *L. pneumophila* (SG1)	67/191 (35.08%)	1457.06 ± 12,969.76		
	Only non-*pneumophila* *Legionella* species	41/191 (21.47%)	132.87 ± 584.01		
	Both species	83/191 (43.46%)	4136.19 ± 22,787.28		
A	Total *Legionella*	102/127 (80.31%)	7434.23 ± 31,459.66	49.16 ± 2.49 (33.27–54.10)	13.23 ± 4.84 (1.00–25.00)
	Only *L. pneumophila* (SG1)	26/102 (25.49%)	2550.13 ± 17,709.16		
	Only non-*pneumophila* *Legionella* species	26/102 (25.49%)	229.60 ± 783.67		
	Both species	50/102 (49.02%)	6476.85 ± 30,675.22		
B	Total *Legionella*	48/122 (39.34%)	1066.80 ± 4520.13	49.40 ± 2.48 (39.85–65.00)	11.85 ± 4.56 (0–25.00)
	Only *L. pneumophila* (SG1)	23/48 (47.92%)	153.75 ± 340.35		
	Only non-*pneumophila* *Legionella* species	9/48 (18.75%)	11.64 ± 39.15		
	Both species	16/48 (33.33%)	2546.06 ± 6985.17		
C	Total *Legionella*	41/58 (70.69%)	333.93 ± 613.4	49.72 ± 3.07 (32.50–57.00)	10.89 ± 6.60 (0–22.50)
	Only *L. pneumophila* (SG1)	18/41 (43.90%)	263.54 ± 679.57		
	Only non-*pneumophila* *Legionella* species	6/41 (14.63%)	34.15 ± 126.99		
	Both species	17/41 (41.46%)	174.70 ± 340.95		

**Table 5:** *Legionella* mean concentration comparison in hospital complex and within buildings.

Building	*Legionella* Comparisons	Mann–Whitney Test *p*–Value	*Legionella* Comparisons	Mann–Whitney Test *p*-Value
Hospital complex	Both species ≠ only *L. pneumophila*	7.48 × 10^−3^ *	Both species > only *L. pneumophila*	3.74 × 10^−3^ *
	Both species ≠ only non-*pneumophila Legionella* species	4.28 × 10^−8^ *	Both species > only non-*pneumophila Legionella* species	2.14 × 10^−8^ *
	only *L. pneumophila* ≠ only non-*pneumophila Legionella* species	1.54 × 10^−3^ *	Only *L. pneumophila* > only non-*pneumophila Legionella* species	7.71 × 10^−4^ *
A	Both species ≠ only *L. pneumophila*	1.95 × 10^−4^ *	Both species > only *L. pneumophila*	9.74 × 10^−5^ *
	Both species ≠ only non-*pneumophila Legionella* species	3.47 × 10^−5^ *	Both species > only non-*pneumophila Legionella* species	1.74 × 10^−5^ *
	only *L. pneumophila* ≠ only non-*pneumophila Legionella* species	0.8696		
B	Both species ≠ only *L. pneumophila*	0.6593		
	Both species ≠ only non-*pneumophila Legionella* species	0.03 *	Both species > only non-*pneumophila Legionella* species	0.015 *
	only *L. pneumophila* ≠ only non-*pneumophila Legionella* species	8.07 × 10^−4^ *	Only *L. pneumophila* > only non-*pneumophila Legionella* species	0.015 *
C	Both species ≠ only *L. pneumophila*	0.97		
	Both species ≠ only non-*pneumophila Legionella* species	3.70 × 10^−3^ *	Both species > only non-*pneumophila Legionella* species	1.85 × 10^−3^ *
	only *L. pneumophila* ≠ only non-*pneumophila Legionella* species	2.75 × 10^−3^ *	only *L. pneumophila* > only non-*pneumophila Legionella* species	1.85 × 10^−3^ *

* *p* ≤ 0.05.

**Table 6:** Matrix correlation between *L. pneumophila*, non-*pneumophila Legionella* species, temperature, and disinfectant (Spearman’s rho test).

Building	*L. Pneumophila* vs. Non-*Pneumophila Legionella* Species	*L. Pneumophila* vs. Temperature	*L. Pneumophila* vs. Disinfectant	Non-*Pneumophila Legionella* Species vs. Temperature	Non-*Pneumophila Legionella* Species vs. Disinfectant
Hospital complex	r_s_ = 0.33 *p* = 1 × 10^−5^ *	r_s_ = −0.29 *p* = 1 × 10^−5^ *	r_s_ = −0.20 *p* = 4 × 10^−4^ *	r_s_ = −0.33 *p* = 1 × 10^−5^ *	r_s_ = −0.07 *p* = 0.22
A	r_s_ = 0.19 *p* = 0.031 *	r_s_ = −0.36 *p* = 1 × 10^−5^ *	r_s_ = −0.10 *p* = 0.25	r_s_ = −0.45 *p* = 1 × 10^−5^ *	r_s_ = −0.096 *p* = 0.28
B	r_s_ = 0.42 *p* = 1 × 10^−5^ *	r_s_ = −0.27 *p* = 2.80 × 10^−3^ *	r_s_ = −0.35 *p* = 1 × 10^−5^ *	r_s_ = −0.28 *p* = 2.10 × 10^−3^ *	r_s_ = −0.14 *p* = 0.12
C	r_s_ = 0.14 *p* = 0.31	r_s_ = −0.35 *p* = 6.50 × 10^−3^ *	r_s_ = −0.24 *p* = 0.075	r_s_ = −0.30 *p* = 0.024 *	r_s_ = −0.15 *p* = 0.25

* *p* ≤ 0.05.

## Supplementary Materials

The following are available online at https://www.mdpi.com/1660-4601/17/22/8662/s1, Figure S1: Risk map of *Legionella* spp. distribution in the Hospital: Building A (blue square), B (yellow square) and C (green square) (floors from −1 to 2), Figure S2: Risk map of *Legionella* spp. distribution in the Hospital: Building A (blue square), B (yellow square) and C (green square) (floors from −1 to 2), Figure S3: Correlation matrix of Hospital Complex: *Legionella* mean concentration *vs* temperature and disinfectant (*** *p* value < 0.0001), Figure S4: Correlation matrix of Building A: study of relationship between *L. pneumophila*, *Legionella* non-*pneumophila* species, temperature and disinfectant (* *p* value < 0.05, *** *p* value < 0.0001), Figure S5: Correlation matrix of Building B: study of relationship between *L. pneumophila*, *Legionella* non-*pneumophila* species, temperature and disinfectant (** *p* value < 0.01, *** *p* value < 0.0001), Figure S6: Correlation matrix of Building C: study of relationship between *L. pneumophila*, *Legionella* non-*pneumophila* species, temperature and disinfectant (* *p* value 0.05–0.1, * *p* value < 0.05, ** *p* value < 0.01), Table S1: Physical and chemical parameters of water in Hospital aqueduct, tap water output and hot water return lines.
