# Supplementary material for: How Molecular Typing Can Support Legionella Environmental Surveillance in Hot Water Distribution Systems: A Hospital Experience
Source: Int J Environ Res Public Health. 2020 Nov 21;17(22):8662. doi: 10.3390/ijerph17228662 (PMC7700474; doi:10.3390/ijerph17228662)
Supplement: Supplementary file 1 [file ijerph-17-08662-s001.pdf]

SUPPLEMENTARY FILES

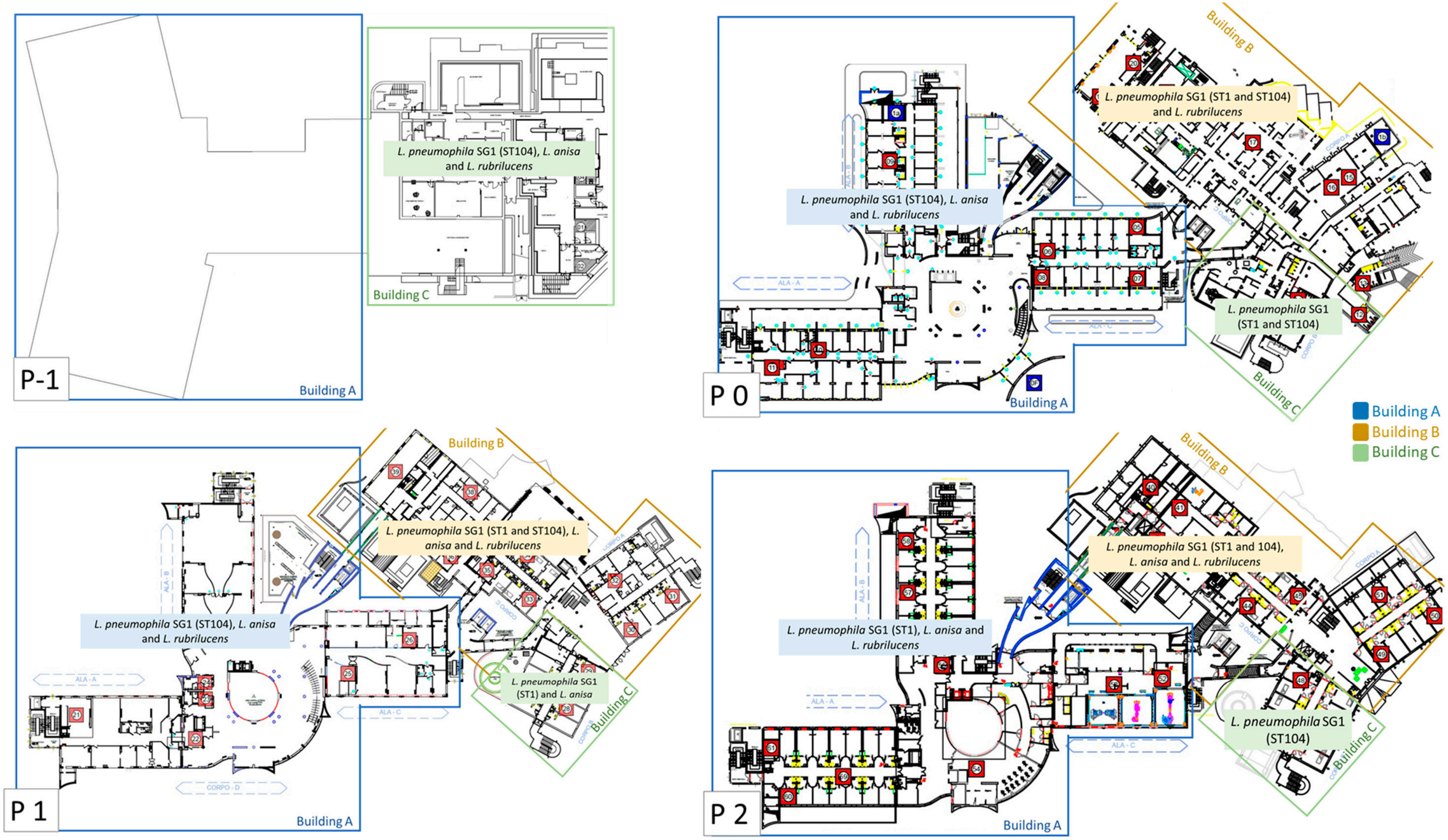

Figure S1: Risk map of *Legionella* spp. distribution in the Hospital: Building A (blue square), B (yellow square) and C (green square) (floors from -1 to 2)

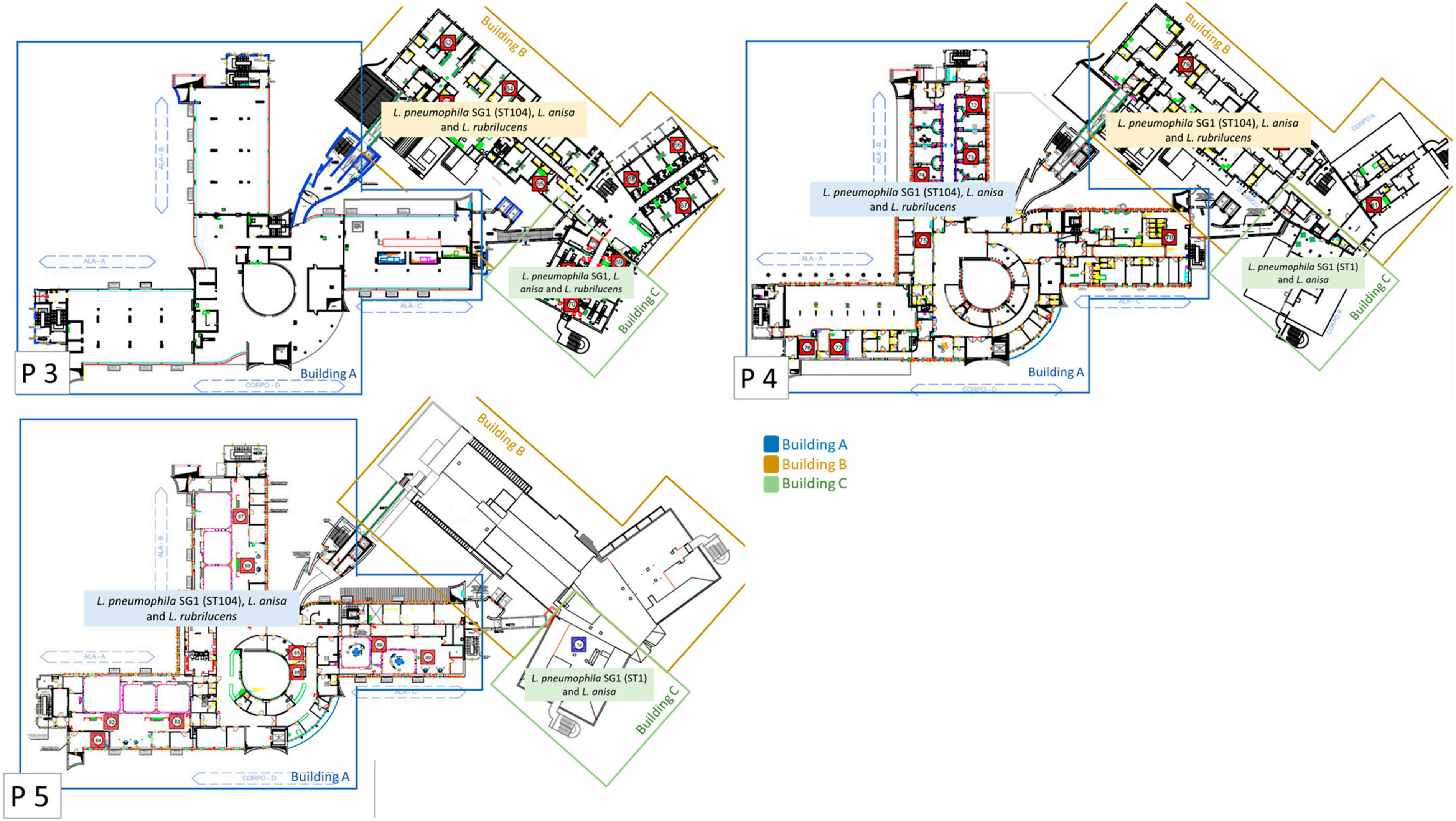

Figure S2: Risk map of *Legionella* spp. distribution in the Hospital: Building A (blue square), B (yellow square) and C (green square) (floors from -1 to 2)

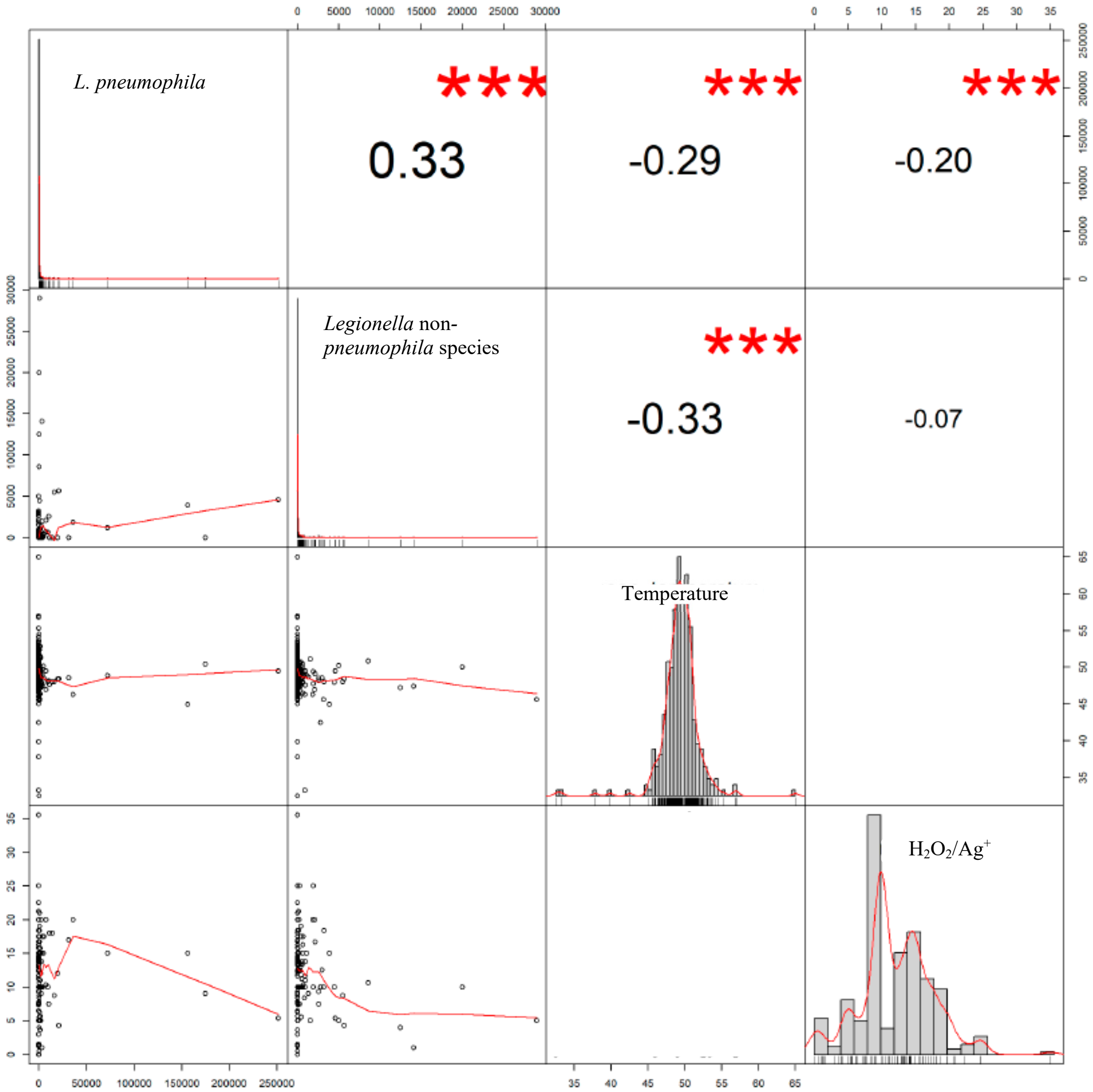

Figure S3: Correlation matrix of Hospital Complex: *Legionella* mean concentration *vs* temperature and disinfectant (***p value < 0.0001)

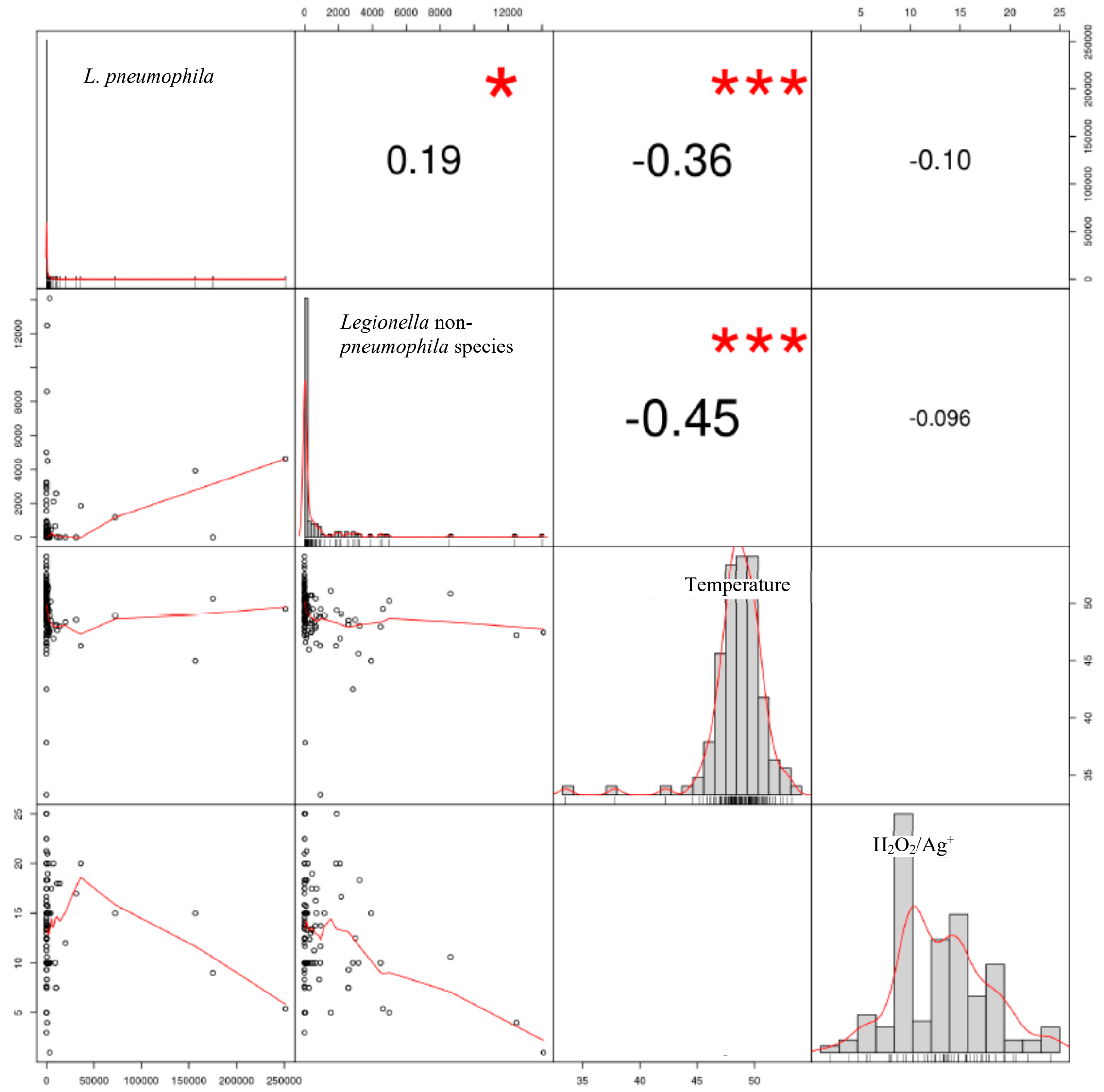

Figure S4: Correlation matrix of Building A: study of relationship between *L. pneumophila*, *Legionella* non-*pneumophila* species, temperature and disinfectant (*p value < 0.05, ***p value < 0.0001)

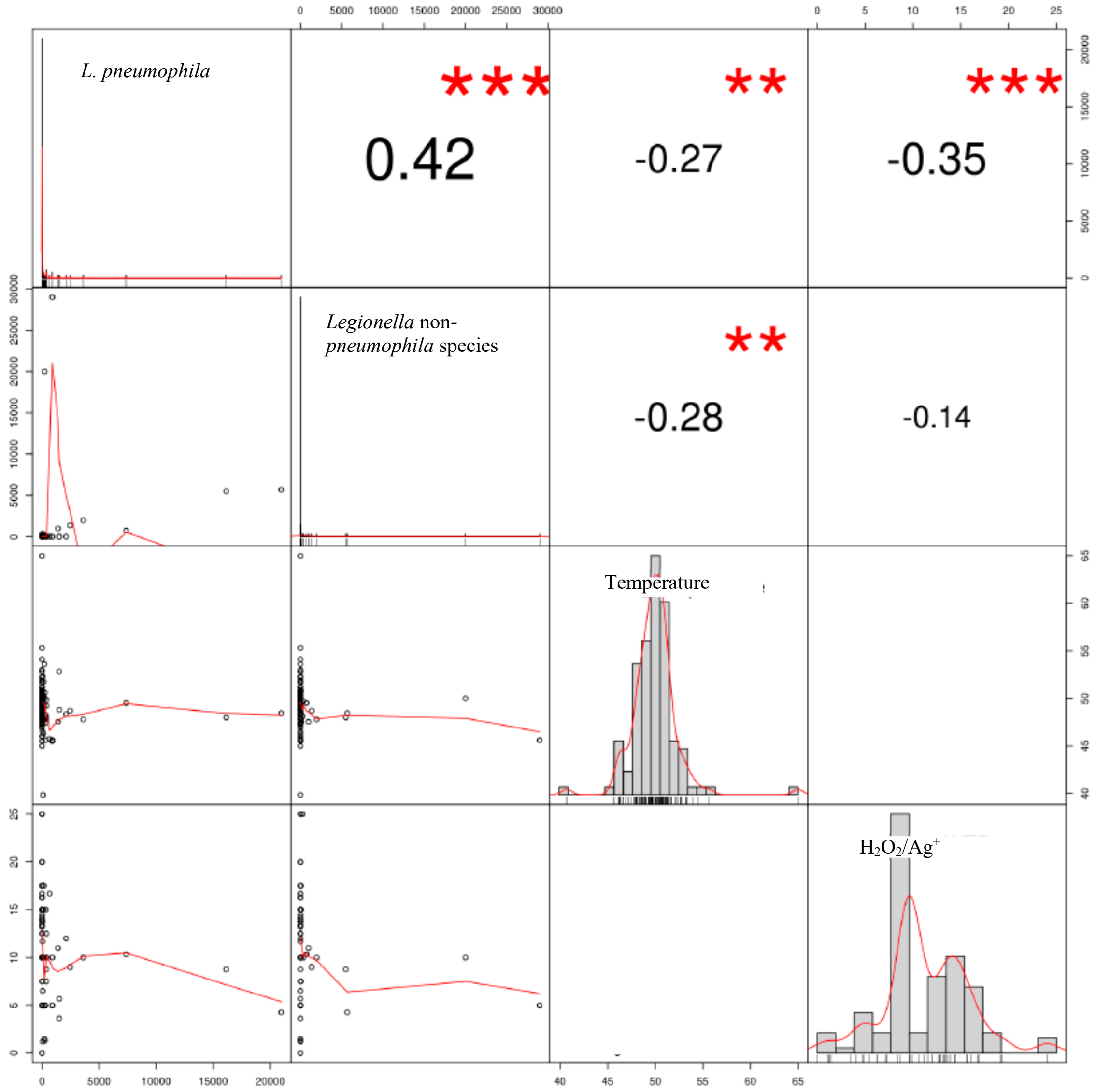

Figure S5: Correlation matrix of Building B: study of relationship between *L. pneumophila*, *Legionella* non-*pneumophila* species, temperature and disinfectant (**p value < 0.01, ***p value < 0.0001)

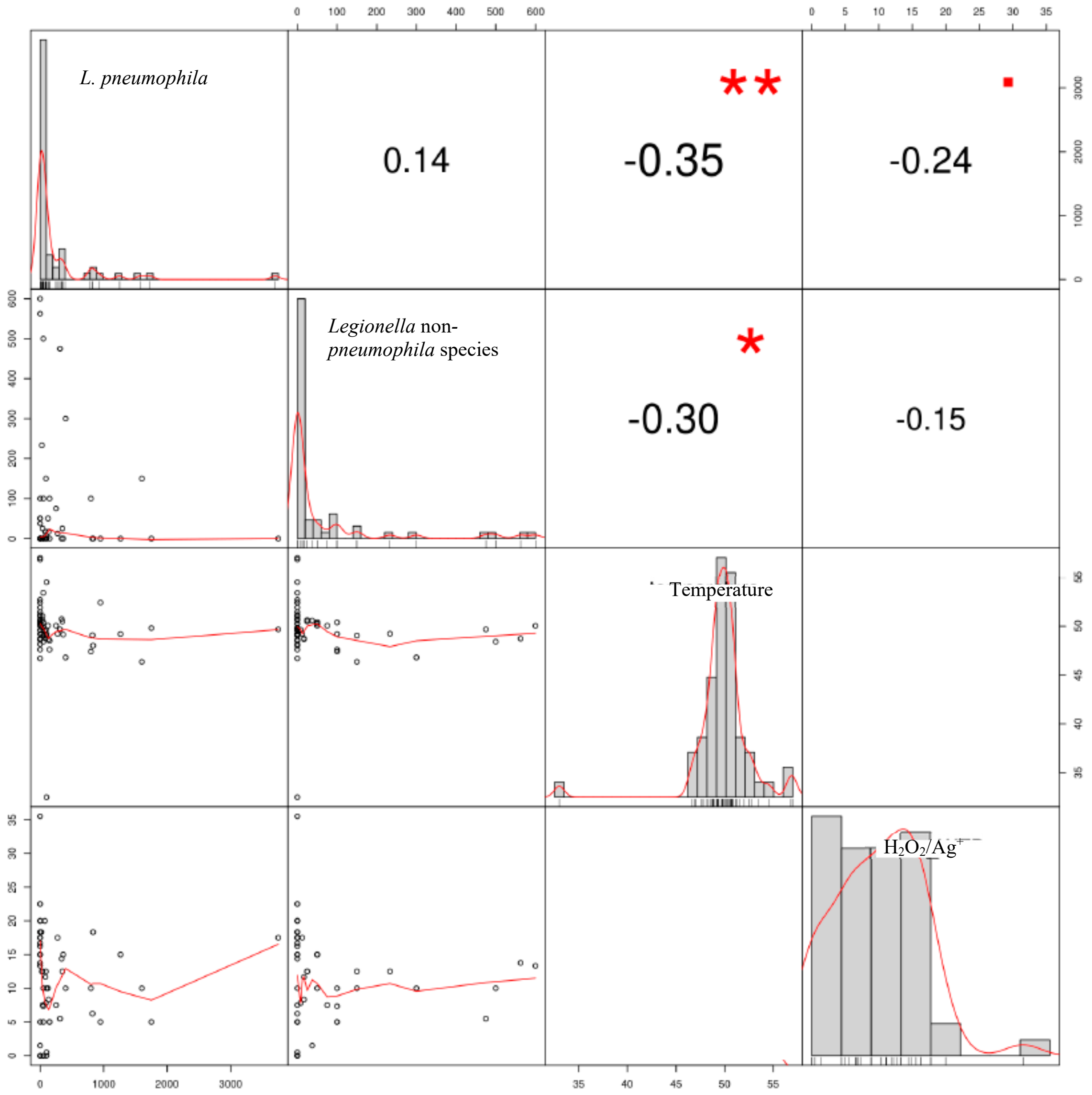

Figure S6: Correlation matrix of Building C: study of relationship between *L. pneumophila*, *Legionella* non-*pneumophila* species, temperature and disinfectant (▪p value 0.05-0.1, *p value < 0.05, **p value < 0.01)

Table S1: Physical and chemical parameters of water in Hospital aqueduct, tap water output and hot water return lines

**Parameters**		**pH**	**Hardness**	**Conductivity**	**Turbidity**	**Total iron**	**Total phosphorus**	**Silver**	**Temperature**	**Peroxide**
**U.M.**			° f	µS/cm	NTU	mg/L	mg/L P_2O_5	µg/L	° C	mg/L
**Sampling points (mean ± SD)**	**Water reservoir**	7.89 ± 0.33	12.08 ± 3.98	399.21 ± 34.21	0.37 ± 0.06	<0.04	<0.3	<3	17.80 ± 1.97	not detected
	**Water Output outlets**	7.83 ± 0.28	11.15 ± 4.03	402.35 ± 33.82	0.41 ± 0.08	<0.04	<0.2	<3	13.72 ± 4.41	not detected
	**Hot water return line (Building A)**	7.93 ± 0.26	12.23 ± 3.04	415.87 ± 40.86	0.38 ± 0.09	<0.04	3.17 ± 1.27	<3	49,50 ± 0,94	13,70 ± 4,03
	**Hot water return line (Building B)**	7.95 ± 0.25	12.73 ± 3.55	419.32 ± 41.56	0.50 ± 0.29	<0.04	3.25 ± 1.90	<3	49,84 ± 0,56	12,24 ± 5,90
	**Hot water return line (Building C)**	7.85 ± 0.17	12.35 ± 2.22	421.22 ± 28.76	0.91 ± 0.87	<0.04	1.58 ± 0.78	<3	49,64 ± 0,84	10,48 ± 5,07
